# Isolated Osteochondral Autograft Mosaicplasty for In Situ Fixation of Unstable Osteochondritis Dissecans in Skeletally Mature Patients

**DOI:** 10.1016/j.eats.2025.103972

**Published:** 2025-11-29

**Authors:** Stephanie O’Brien, Peter S.E. Davies, Rebecca Rogers, Alistair I.W. Mayne, Peter Edwards, Jay Ebert, Peter A. D’Alessandro

**Affiliations:** a Orthopaedic Research Foundation of Western Australia, Perth, Western Australia; b Department of Trauma and Orthopaedics, Fiona Stanley and Fremantle Hospitals. Group, South Metropolitan Health Service, Perth, Western Australia; c The Robert Jones and Agnes Hunt Orthopaedic Hospital, Oswestry, U.K.; d Craigavon Area Hospital, Craigavon, Northern Ireland; e School of Allied Health, Curtin University, Perth, Western Australia; f School of Human Sciences (Exercise and Sport Science), University of Western Australia, Perth, Western Australia; g HFRC Rehabilitation Clinic, Perth, Western Australia; h Division of Surgery, Medical School, University of Western Australia, Perth, Western Australia

## Abstract

Osteochondritis dissecans of the adult knee may result in unstable lesions that cause pain and mechanical symptoms. These often require surgery due to the failure of conservative management. Surgical techniques aim to retain the fragment where possible. Mosaicplasty for in situ fixation provides mechanical stability and a biological stimulus without the need for additional hardware. This all-arthroscopic technique uses osteochondral autograft plugs harvested from a nonweightbearing part of the knee to bridge through the lesion into the subchondral bone. Graft diameter and length can be tailored to the lesion and patient. Postoperatively, patients commence early range-of-motion exercises and follow a graduated weightbearing protocol.

Osteochondritis dissecans (OCD) is an idiopathic condition affecting cartilage and subchondral bone. It is a spectrum of pathology, which may lead to unstable osteochondral fragments that become detached within the joint. The underlying etiology is poorly understood.^1^

Knee OCD can be divided into juvenile and adult forms. Most juvenile OCD lesions occur in the posterolateral aspect of the medial femoral condyle, but higher rates of lateral disease have been reported in adults, possibly representing refractory atypical juvenile disease that has persisted into adulthood.^2^ Most juvenile lesions are stable and heal without long-term sequelae, but most adult lesions are unstable and cause pain, effusions, and mechanical symptoms. When unstable lesions become detached, they create loose bodies, chondral defects, and irregular joint surfaces, which may lead to osteoarthritis.^1^^,^^2^ Unstable lesions are often treated surgically; the treatment largely depends on whether it is possible to retain the unstable fragment. Where the fragment is not salvageable, techniques may be required to restore the lost articular surface, including traditional mosaicplasty or other chondral restoration procedures, including autologous chondrocyte implantation and osteochondral allograft.^3^^,^^4^ Where salvage is possible, traditional methods have included the use of bioabsorbable pins or metal screws to achieve adequate fixation and stability. These methods do not provide a large biological stimulus and leave defects in the chondral surface. To provide a biological stimulus, intact cartilage must be disrupted to lift the fragment and gain access to the underlying sclerotic subchondral bone for stimulation or bone grafting. An alternative approach is to use mosaicplasty for in situ fixation of the fragment, which provides a fixation of similar tensile strength to screws, and a significant biological stimulus via drilling through the subchondral bone and implantation of an autograft plug directly across the sclerotic rim.^5^^,^^6^ Although this technique was first described over 20 years ago, its use is not widespread.^3^^,^^4^^,^^6^ We find it to be a useful treatment option in these challenging cases.

### Indications

Appropriate patients include those with nondisplaced unstable OCD lesions (Fig 1) with mechanical symptoms that have failed conservative measures. This technique is not appropriate for patients with radiographic joint space narrowing or those who are unable to follow the rehabilitation protocol. Knee stability should be concomitantly achieved through ligament reconstruction, while coronal plane malalignment of the limb toward the affected side should be corrected with osteotomy before or at the time of in situ fixation.

**Fig 1:**
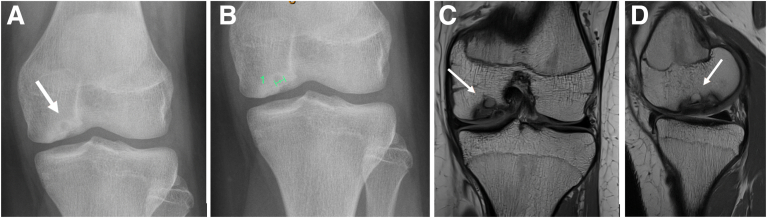
(A) Plain anteroposterior radiograph showing irregular distal medial femoral joint line, suspicious of osteochondritis dissecans (OCD), with the area of concern demarcated. These appearances warrant further investigation with magnetic resonance imaging (MRI). (B) Plain lateral radiograph taken preoperatively of a symptomatic patient showing a demarcated OCD lesion. (C, D) T1-weighted coronal and sagittal MRI sequence showing a medial femoral condyle OCD unstable lesion prior to any surgical intervention.

## Surgical Technique

### Preoperative Planning

**Fig 2:**
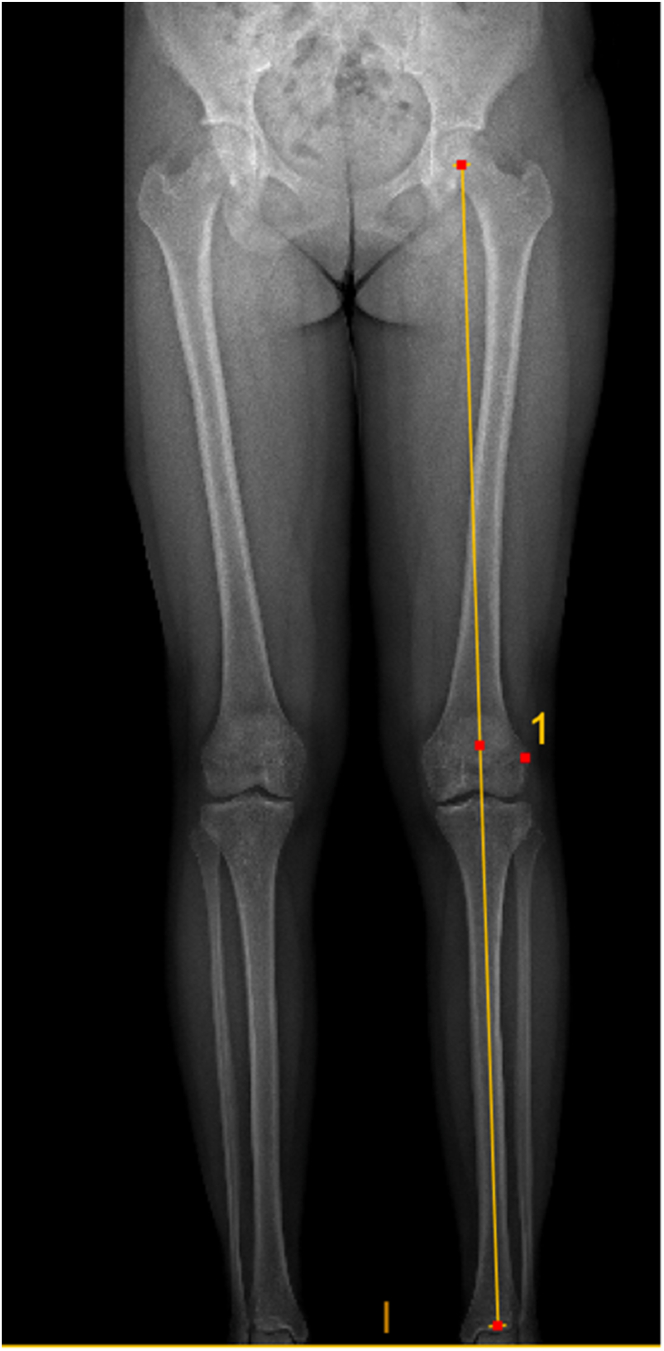
EOS scan showing neutral coronal plane alignment preoperatively.

Preoperative imaging includes plain x-ray, magnetic resonance imaging (MRI) scan, and coronal plane alignment (EOS scan [EOS Imaging, Paris, France]; Fig 2) for all patients. Patients with evidence of bone loss are further referred for a computed tomography scan.

### Diagnostic Arthroscopy

Under a general anesthetic with the use of a high thigh tourniquet, a diagnostic knee arthroscopy is performed with vertical anterolateral and anteromedial portals. The osteochondral lesion is assessed for size, location, and stability. Suitability for in situ fixation with mosaicplasty is confirmed.

### Graft Harvest

A reusable graft site mosaicplasty kit with multiple size options (2.7 mm, 3.5 mm, 4.5 mm, 6.5 mm, and 8.5 mm) (Mosaicplasty System; Smith & Nephew) is opened in the operating theater. The lesion remains in situ and is not removed to debride the underlying sclerotic bone. The surgeon identifies the diameter and quantity of plugs that will be required. A consumable harvesting kit corresponding to the diameter of plugs required is opened (Mosaicplasty Harvesting; Smith & Nephew). We find that 4.5-mm or 6.5-mm plugs are the most useful sizes, and we harvest plugs using an all-arthroscopic technique. The length of the plugs should be at least 10 mm long but generally range from 15 to 20 mm. Plugs should bypass the sclerotic bed of the OCD, and this should be measured preoperatively on MRI to ensure adequate plug length intraoperatively. Open procedures should be considered when there is difficulty accessing the site arthroscopically.

**Fig 3:**
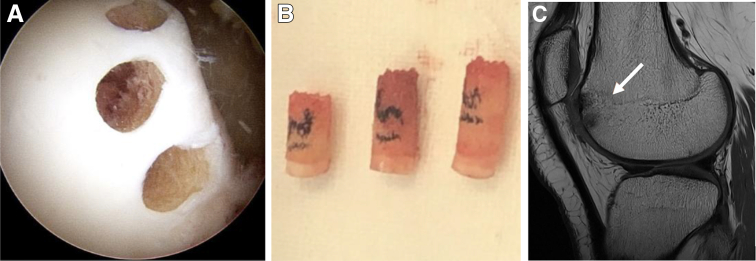
(A) Patient in the supine position. Arthroscopy of the left knee through the anterolateral portal, with view of the harvest site of 3 osteochondral autograft plugs from the lateral trochlea. (B) Intraoperative image of 3 osteochondral autograft plugs of various lengths visualized on the prep table following harvest, ready for reimplantation to the site of the osteochondritis dissecans lesion. (C) Sagittal T1-weighted magnetic resonance imaging of the mosaicplasty donor site showing restoration of bone and cartilage postoperatively.

The routine harvest site is from the nonweightbearing portion of the lateral or medial trochlea (Fig 3A) or alternatively around the femoral notch. There should be a few millimeters between each harvest site, and the confluence of the tunnels should be avoided. The graft is harvested to the appropriate depth, and then it is retrieved and measured (Fig 3B). The harvest site is not backfilled, and all of our patients have shown restoration of harvest-size bone/cartilage by the time of the 4- to 6-month MRI scan (Fig 3C).

### Graft Implantation

**Fig 4:**
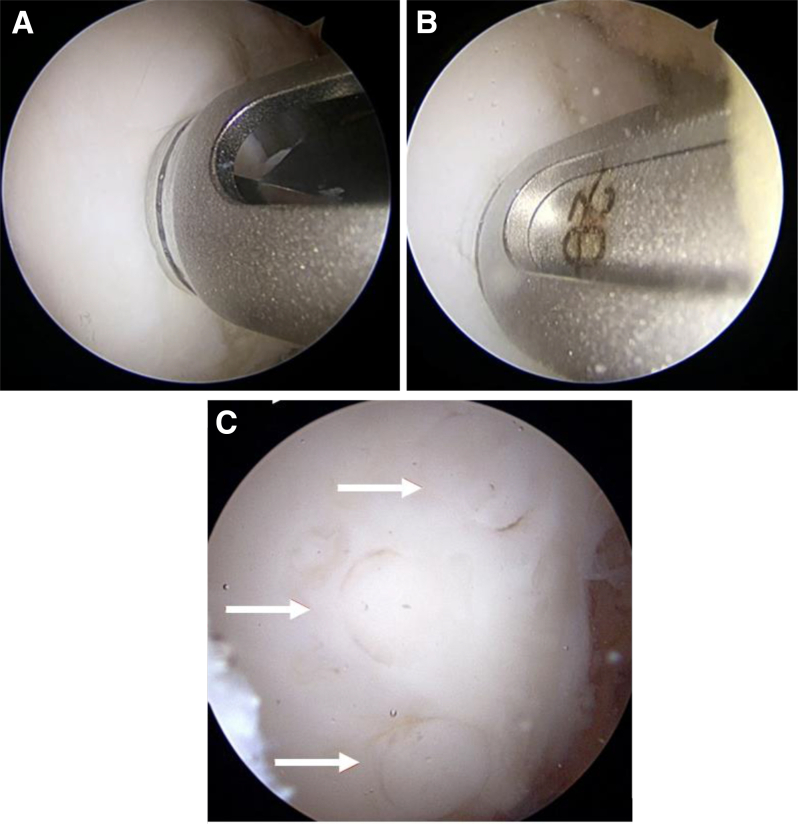
(A) Patient in the supine position. Arthroscopy of the left knee through the anteromedial portal prior to drilling into the recipient site through the cannulated guide to allow for implantation of mosaicplasty plugs. (B) Patient in the supine position. Arthroscopy of the left knee through the anteromedial portal view of the dilator placed down the cannulated guide without moving position, readying the recipient site for the mosaicplasty graft plug to be inserted. (C) Patient in the supine position. Arthroscopy of the left knee through the anterolateral portal view of mosaicplasty graft plugs after insertion into the prepped donor site to fit a smooth articular surface.

At the site of the lesion, a drill of corresponding diameter to the plug is drilled to 1 mm longer than the measured plug, and the hole is expanded slightly with a dilator (Fig 4 A and B). This allows easy passage of the graft. The plug length must bypass the osteochondral defect and the underlying cystic subchondral bone. Plugs should be spaced at least 2 mm apart to minimize the risk of fragmentation or plug convergence, compromising press-fit fixation. A cannulated guide is used for drilling, dilation, and passage of the graft. The graft is gently impacted to the correct depth, ensuring a press-fit and a smooth articular surface (Fig 4C). The process is repeated with the desired number of plugs, which is generally between 2 and 4 in our institution. After sufficient fixation is achieved, the knee is cycled to ensure stability of the lesion and appropriate plug height.

### Postoperative Protocol

Postoperatively, patients are managed in a hinged brace nonweightbearing for 2 weeks. Immediate supine range-of-motion exercises within the brace are allowed. At 2 weeks, touch weightbearing is permitted for a further 4 weeks in the brace. Thereafter, a gradual increase to full weightbearing is encouraged out of the brace. A wound check is performed at 2 weeks postoperatively, and clinical reviews are performed at 8 weeks and 4 months postoperatively. At 4 to 6 months postoperatively, a MRI scan is obtained to assess for healing and plug integration. If clinical and radiological assessment at this time point has confirmed healing, escalation of rehabilitation, including return to running and progressive return to sport, is allowed. Repeat scans and slowed rehabilitation are performed if there is inadequate evidence of healing.

## Discussion

The technique shown in this article provides an all-arthroscopic method to treat unstable OCD in the adult patient. The pearls and pitfalls that are mentioned in Video 1 are displayed in Table 1. There are several advantages to this technique compared with traditional methods (Table 2). Ordinarily, surgical management of these lesions would require an open approach with elevation of the fragment (thus damaging the intact peripheral articular cartilage) to access the sclerotic subchondral bone, as well as the use of bone graft if deemed necessary. Following debridement of the subchondral bone, the lesion would be reduced and fixed with hardware involving drilling through the articular cartilage, leaving defects at the site of each screw or pin, with the potential for hardware irritation, loosening, and possible future hardware removal. The presented technique can be performed all-arthroscopically with 2 or more portal-sized incisions. This allows the OCD lesion to remain in situ, preventing disruption to the intact articular cartilage layer, and results in complete coverage of articular cartilage in the affected weightbearing zone. There is a structural autograft crossing the sclerotic rim of the OCD lesion, providing a unique combination of mechanical stability and direct biology with each plug. There is no hardware to be removed in the future. There is elimination of the risk of the fragment being dropped on the floor during surgery and a reduced risk of fragmentation that may occur during open debridement.

**Table 1:** Technique Pearls and Pitfalls When Performing Mosaicplasty for In Situ Fixation of Unstable OCD in Adult Patients

**Pearls**
Carefully plan the required number and length of plugs using MRI preoperatively to ensure adequate coverage and fixation across the cyst/sclerotic rim of OCD.
Plan the diameter and position of plugs carefully with arthroscopic assessment.
Reverse the harvester and use the central core and harvesting “hood” to tap the plug out of the harvester by tapping on the bony (not chondral) surface to minimize damage to cartilage.
Use accessory arthroscopic portals as required to ensure both harvest and insertion are perpendicular to the donor/implantation site.
Turn off the arthroscopy fluid supply when introducing plugs to prevent them from being pushed back out of the introducer.
**Pitfalls**
Be very careful to not drop plugs once harvested.
Use fewer plugs rather than more: plug convergence and/or fragmentation of the lesion is to be avoided.
Avoid convergence of harvest sites for multiple plugs as this may lead to inadequate plug lengths and cause fracture.
Ensure plugs are not too long for the tunnel depth drilled, or plugs may remain proud once impacted.

MRI, magnetic resonance imaging; OCD, osteochondritis dissecans.

**Table 2:** Advantages and Disadvantages of Using Mosaicplasty for In Situ Fixation of Unstable OCD in Adult Patients, Compared With Traditional Methods

**Advantages**
Can be performed all-arthroscopically
Keeps articular cartilage periphery intact
No additional hardware required that may become loose/prominent
No risks of hardware removal in the future
Does not leave defects in the weightbearing surface
Eliminates the possibility of dropping the OCD lesion from the surgical table
Reduced risk of fragmenting the lesion during instrumentation
**Disadvantages**
May fracture plug or harvest inadequate plug
Donor site morbidity, including fracture
Delayed or nonunion of construct

OCD, osteochondritis dissecans.

Potential drawbacks of using this technique may include fracture of the plugs during insertion (which would reduce the construct’s mechanical stability), donor site morbidity (well documented to be acceptable with mosaicplasty and not found to be a concern in our cohort, given complete harvest site restoration), delayed or nonunion of the fragments (which may also still occur using traditional methods), and confluence of the harvest sites, which may cause a fracture or inadequate graft harvest. In certain cases, it may be helpful to convert to an open procedure, especially when first learning the technique. We have found this to be an effective and reproducible technique for the treatment of these challenging cases, with excellent rates of healing and clinical outcomes.

## Disclosures

The authors declare the following financial interests/personal relationships which may be considered as potential competing interests: P.A.D. is a consultant or advisor for 10.13039/100009026Smith & Nephew and Medacta Australia Pty Ltd; has received funding grants from Smith & Nephew, Arthrex, and DePuy Orthopaedics; has received speaking and lecture fees from Smith & Nephew, Medacta Australia Pty Ltd, and Arthrex; has received travel reimbursement from Smith & Nephew, Medacta Australia Pty Ltd, Arthrex, Pune Knee Course, and Thai Orthopaedics Sports Medicine Society. All other authors (S.O., R.R., P.E., J.E., P.S.E.D., A.I.W.M.) declare that they have no known competing financial interests or personal relationships that could have appeared to influence the work reported in this paper.

## Supplementary Data

Video 1 Here, we describe our technique for performing arthroscopic isolated mosaicplasty for in situ fixation of osteochondritis dissecans in the adult patient. This includes the selection of equipment, arthroscopy, careful harvest of osteochondral plugs, identifying the unstable lesion and the site(s) for plug donation, and drilling and insertion of plugs to create a mechanically strong environment with a biological stimulus to promote healing.
